# Anti-inflammatory Approaches to Mitigate the Neuroinflammatory Response to Brain-Dwelling Intracortical Microelectrodes

**DOI:** 10.29245/2578-3009/2018/4.1157

**Published:** 2018-08-03

**Authors:** Hillary W. Bedell, Jeffrey R. Capadona

**Affiliations:** 1 department of Biomedical Engineering, Case Western Reserve University, School of Engineering, 2071 MLK Jr. Drive, Wickenden Bldg, Cleveland OH 44106, USA; 2 Advanced Platform Technology Center, L. Stokes Cleveland VA Medical Center, Rehab. R&D, 10701 East Blvd. Mail Stop 151 AW/APT, Cleveland OH 44106, USA

**Keywords:** Intracortical Microelectrode, Brain, Anti-Inflammatory, Brain-Machine Interface, Neuroinflammation, Glial Response

## Abstract

Intracortical microelectrodes are used both in basic research to increase our understanding of the nervous system and for rehabilitation purposes through brain-computer interfaces. Yet, challenges exist preventing the widespread clinical use of this technology. A prime challenge is with the neuroinflammatory response to intracortical microelectrodes. This mini-review details immunomodulatory strategies employed to decrease the inflammatory response to these devices. Over time, broad-spectrum anti-inflammatory approaches, such as dexamethasone and minocycline, evolved into more targeted treatments since the underlying biology of the neuroinflammation was elucidated. This review also presents studies which examine novel prospective targets for future immunomodulatory targeting.

## Introduction: Brain-dwelling Intracortical Microelectrodes—Uses and Limitations

Intracortical microelectrodes were created as a tool for basic neuroscience research to understand the normal and diseased physiology of the brain for the treatment of neurological disorders, and they have since expanded to become critical components for brain-machine interfacing (BMI)^1–3^. BMI technologies have shown great success in enabling locked-in patients to interact with computers, robotic limbs, and their own electrically driven limbs^3,4^. The recent advances have inspired worldwide enthusiasm resulting in billions of dollars of federal and industrial sponsorships to promote understanding the brain for rehabilitative and preventative medicine applications. Additionally, private philanthropists have also demonstrated excitement in the field by investing in the use of brain interfacing technologies as a means to human augmentation^5,6^. In the future, brain-dwelling microelectrodes could restore patterns of normal brain function in diseased or injured patients through therapeutic modulation of dysfunctional pathways. Thus, implantable microelectrodes in the future can be transformative in not only restoring sensorimotor abilities but also improving the treatment of brain disorders and augmenting human capabilities.

While the promise of these incredible technologies is real, caution must be taken as implications regarding optimal performance and unforeseen side effects following device implantation into the brain are not fully characterized. For example, we recently demonstrated that rats with intracortical microelectrodes implanted in the primary motor cortex exhibited a remarkable 527% increase in the time required to complete a fine motor task^7^. Notably, the increased time to complete fine motor tasks was correlated with persistent damage to the blood-brain barrier, as a result of the neuroinflammatory response.

Despite the incredible enthusiasm for brain interfacing technologies, it is widely understood that microelectrodes for BMI technologies exhibit limited long-term viability where recordings typically fail 6 months to 1 year after implantation, due to multimodal failure mechanisms.^8^ One of the primary causes for this failure is believed to originate from the perpetual inflammatory response following implantation^9,10^. Since the neurodegenerative progression of the disease and injured states are often associated with the progression of cognitive and motor-related disease symptoms, the relative contribution of implanting potentially restorative devices that could actually contribute to the perceived progression of disease-like symptoms remains unclear, and in most cases untested. Therefore, these critical questions need to be answered in respect to both understanding disease progression and the role of interfacing technologies in both treating and propagating disease-like symptoms of deteriorated cognitive and motor abilities.

Fortunately, much work is being done surrounding microelectrode design to reduce the inflammatory response (for review see^8,11^). Although significant progress has been made fabricating ultra-small implants, a universal approach to reduce inflammation regardless of the device decreases the need for design restrictions for successful tissue integration. For example, larger brain-dwelling electrodes are necessary to reach deep brain structures such as nucleus accumbens^12^. Additionally, in order to input/output more degrees of freedom from these neural prostheses, many electrode sites are needed resulting in designs with greater surface area^13^. Larger electrodes result in more foreign material in the brain disrupting tissue; the more invasive the implant, the more tissue is damaged during implantation. Ideally, lessons learned about the neuroinflammatory response to smaller intracortical microelectrodes can be applied to future applications of any brain-dwelling electrode, regardless of size or design. The factors that drive the inflammatory response must be targeted to reduce neuronal dysfunction and damage, to lead to improved signal for any application using implantable microelectrodes to ensure chronic, high-fidelity function.

An active immunomodulatory approach should afford the resolution of the damage caused during the implantation of the microelectrode, while also curtailing the chronic neurodegenerative aspects of the neuroinflammatory response to enhance the long-term function of the device. Furthermore, an immunomodulatory approach should also work in combination with innovative design strategies to combat the foreign body response, as no one solution can adapt as fast as the dynamic temporally facilitated biological response. In addition to modifications in the design of the probe to mitigate the inflammatory response, there are also biological and materials-based approaches undertaken to reduce neuroinflammation to chronically implanted intracortical microelectodes (reviewed in^8^). In this mini-review, however, we focus on only direct immunomodulatory approaches applied to reduce the inflammatory response and improve the function of implantable intracortical microelectrodes, and we provide our current vision of the leading prospects moving forward.

## Discussion

The following sections will discuss leading active anti-inflammatory approaches to mitigate microelectrode-induced neuroinflammation.

### Steroids: Dexamethasone (DEX)

The first method to directly modulate the immune system after implantation of microelectrodes was the use of systemic injection of dexamethasone (DEX), commonly used for the treatment of multiple sclerosis in the clinic. DEX is a synthetic glucocorticoid that induces pleiotropic anti-inflammatory functions through cellular glucocorticoid receptors. Most cells, including microglia, express receptors for glucocorticoids such as dexamethasone^14^.

The first group to report the use of DEX to minimize neuroinflammation in response to intracortical microelectrodes peripherally injected DEX (200 mg/kg) in rats daily for six days beginning the day of surgery. The systemic administration of DEX led to decreased astrocytic response at 1 and 6 weeks post-implantation^15^. However, the same systemic repeated administration of DEX at a lower dose (200 μg/kg) in rats, led to a “transient increase” in both microglial/macrophage responses and laminin deposition which was interpreted by the authors as a response to injury, repair, and/or angiogenesis^16^. Since high systemic delivery of steroids can have serious side effects, DEX has also been incorporated into different probe coatings: poly(ethyl-vinyl) acetate, nitrocellulose, carbon nanotubes, and poly(lactic-co-glycolic acid) nanoparticles within alginate hydrogel matrices^17,18^. Each of the studies delivering DEX locally examined different markers of neuroinflammation. Benefits common to all the studies included decreased astrocytic response, reduced microglial/macrophage activity, mitigated neuronal loss, and minimized chondroitin sulfate proteoglycan expression^15,19–21^_._

DEX has also been shown to improve the functional recording performance of intracortical microelectrode. For example, DEX was loaded into electrospun biodegradable nano-fibers followed by an alginate hydrogel encapsulation to allow for long-term release. Poly (3,4-ethylene dioxythiophene) (PEDOT) was then electrochemically polymerized on the electrode sites within the scaffold. The composite coating with PEDOT elicited decreased impedance compared to non-PEDOT coated electrodes and allowed for higher applied charge density than traditional electrodes^22^. DEX incorporated into nanoparticle embedded coatings reduced impedance compared to no-drug controls by 25%, likely due to the demonstrated decreased tissue response^23^. Zhong and colleagues were able to release DEX from nitrocellulose coatings for 16 days *in vitro.* However, once the drug is depleted from the probe material, it is currently not possible to reload the material with the drug, Furthermore, acute strategies to mitigate the inflammatory response have not been completely successful at more chronic time points^24,25^. Currently, continuous local delivery of a drug such as DEX can be delivered *in vitro* on a 1.5 mm metal neural probe using microfluidic technology^26^. As the development of this technology progresses to incorporate a microfluidic delivery system into smaller, functional, neural probes, local continuous delivery of DEX can be a promising strategy in a larger multifaceted anti-inflammatory approach.

### Antibiotics

Another broad-spectrum drug that has been investigated to decrease neuroinflammation associated with microelectrode implantation is minocycline. Minocycline is a semi-synthetic tetracycline shown to be neuroprotective in brain and spinal cord injury^27^. Rennekar and colleagues were the first group to use minocycline to improve neural recordings from intracortical microelectrodes. The team dissolved minocycline-HCl in the drinking water of experimental rats for 2 days prior to probe implantation through 5 days post-implantation. It was estimated that the rats received about 4 mg minocycline per day (13–20 mg kg^−1^), based on water consumption. After 6 days, the group receiving minocycline yielded significantly higher signal to noise ratios (SNR) and the percentage of active channels detecting neural activity was higher than the control group. Both metrics for improved recording quality were maintained through 4 weeks post-implantation (study completion). Additionally, glial scarring was reduced with minocycline administration at both of the time points in which histology was examined, 1 and 4 weeks post-implantation^28^. Unfortunately, no report of neuronal density, known to die back around the implants, was provided in Rennekar’s study.

An earlier study had previously shown that to achieve neuroprotective effects, minocycline must be present by the neurons at much higher levels^29,30^. Thus, Zhang *et al.* incorporated minocycline into thin film coatings on oxidized silicon, a common material for neural electrodes^31^. The thin film coatings allow for increased loading and local, sustained release of higher concentrations of minocycline (over 46 days). In preliminary *in vitro* testing, minocycline incorporated in thin film coatings were able to elicit neuroprotective activity similar to controls dispersed directly into the culture media. Although these studies were conducted *in vitro*, the results demonstrated promise for the extended delivery of minocycline via neural probes. Additionally, recent work showed that minocycline delivered through microfluidic channels fabricated within an implanted neural probe led to reductions in microglial reactions between 200–400 μm from the probe interface^32^. Such distances are unlikely to impact neural recording quality. Interestingly, minocycline delivery had no effect on astrocyte density, inconsistent with the original findings from Rennekar *et al.* Similarly, in concordance to the previously mentioned studies, Hayn *et al.* (2017) administered minocycline in a single local dose (20 μg/μL) via cannula implantation in the motor cortex of rats and found decreased neuronal death and anti-inflammatory effects with improved motor function^33^. Overall, these studies demonstrate promise for minocycline to mitigate the neuroinflammatory response around implanted microelectrodes.

However, long-term dosing of minocycline begets an increased risk of adverse events—including hyperpigmentation of the skin and other organs^34,35^. Minocycline possesses an increased chance of serious adverse events relative to other tetracyclines^35^. Thus, minocycline could be part of a multi-faceted approach to reduce initial neuroinflammation, but less risky alternative therapies are needed for chronic applications.

Because of an increased understanding of the biological response to microelectrode implantation, more specific immunomodulatory approaches are being utilized to mitigate the inflammatory response and improve the chronic performance of these devices.

### Mitigating the Glial Response

Both microglia and astrocytes contribute to the biological response affecting electrode function. Thus, an effective way to modulate the immune response would be to mitigate the glial response. The Bellamkonda group utilized alpha-melanocyte stimulating hormone (MSH) (an endogoneous tridecapetide) to target the microglial response^36^. Alpha-MSH has been shown to inhibit both nitric oxide and pro-inflammatory cytokines produced by activated microglia—both of which are detrimental to neuronal health^37^. In their study, the Bellamkonda group coupled the Alpha-MSH peptide to silicon single shank planar microprobes and implanted the probes into the motor cortex of rats. The astrocytic and activated microglial response was examined via histology at 1 and 4 weeks post-implantation. At both time points, the activated microglial response was significantly decreased compared to a non-coated control probe. There was no notable difference in astroglial scarring at 1 week, but at 4 weeks post-implantation significantly less scarring was present^36^.

Purcell *et al.* administered flavopiridol to rats implanted with Michigan-style single shank silicon multi-channel intracortical electrodes^38^. Flavopiridol arrests progression into the cell cycle, yet, re-entry into the cell cycle has been demonstrated in glial activation as shown by upregulation of cell-cycle components^39^. Thus, flavopiridol was hypothesized to lead to decreased glial activation and improved intracortical microelectrode recording performance. However, Purcell *et al.* were unable to demonstrate improved neural recording or decreased presence of glial cells (astrocytes and microglia combined)^38^.

Another immunomodulatory approach that has been explored was to use a cytokine receptor antagonist to reduce neuroinflammation. Interleukin- 1 receptor antagonist (IL-1Ra) has been used in human clinical trials for rheumatoid arthritis treatment and is endogenously released by microglia to facilitate neuroprotection^40,41^. Neural probe coatings incorporated with IL-1Ra have been successfully used to improve neuronal survival and reduce glial scarring as long as four weeks post-implantation^42,43^. Unfortunately, no information has been reported to date about either chronic time points or the effects on recording performance.

### Targeting the Inflammasome

Given the role of the inflammasome in brain injury models, targeting the players of this construct can be pivotal in reducing neuroinflammation following intracortical microelectrode implantation. The inflammasome is an innate immune protein complex. The inflammasome activates caspase-1, an enzyme which allows a pivotal pro-inflammatory cytokine, IL- β, to achieve its active form. IL-β has been shown to be significantly upregulated around intracortical probes and plays a role in blood-brain barrier (BBB) dysfunction^11,44^. Kozai *et al.* used a knock-out mouse model to demonstrate caspase-1 as a promising immunomodulatory target for improving chronic single-unit recordings by intracortical microelectrodes implanted in the visual cortex of mice^45^. Data obtained by Kozai *et al.* suggested that pharmacologic interventions which target the components and downstream players of the inflammasome can yield more stable chronic neural recordings. Future studies could entail using a caspase inhibitor such as VX-765 or Ac-YVAD-cmk, both of which have demonstrated neuroprotection in various models^46–48^.

### Targeting Blood-derived Cells

Blood-derived cells have been shown to be major contributors to the inflammatory response of brain-dwelling intracortical microelectrode, despite the perception of the immune privileged brain^49,50^. Thus, a recently developed hypothesis is that immunomodulatory strategies that limit blood-derived immune cells from the area within the brain, surrounding the implanted neural electrodes, could mitigate the inflammatory response and concurrently improve recording performance. Targeting monocyte chemoattractant protein-1 (MCP-1) using a knock out mouse model was found to decrease the inflammatory response to intracortical microelectrode implantation^51^. MCP-1 is a chemoattractant which attracts monocytes to areas of inflammation, such as the response to a neural electrode in the brain. Although this study did not directly evaluate reductions to blood-derived cells, they found that MCP-1 knock-out mice reduced BBB leakage, microglia/macrophage response, and astrocytic response, all leading to an increased neuronal density at acute and chronic time points. Notably, the improved neuroinflammatory response was recapitulated with pharmacological inhibition of MCP-1 via daily intraperitoneal injections^51^. It will be critical for future studies to also investigate the impact on neural recordings.

### Pattern Recognition Receptors

Given the presence of blood and cellular damage present at the tissue-device interface after implantation, targeting cellular receptors that recognize damage and initialize the production of pro-inflammatory cytokines promises to reduce the inflammatory response and improve intracortical microelectrode performance. Pattern recognition receptors detect cellular damage and blood proteins and are found on microglia, neurons, astrocytes, and blood-derived macrophages present at the probe interface. Through the use of knock-out mouse models and a small-molecule inhibitor, we have previously shown that targeting the toll-like receptor (TLR)/cluster of differentiation 14 (CD14) pathways can improve both acute and chronic microelectrode performance^49,52^. Thus, pattern recognition receptors are amenable targets to improve neural recording from brain-dwelling intracortical microelectrodes, yet appropriate dosing regimens for optimal wound healing and anti-inflammatory responses remains to be determined.

## Conclusion and Perspectives

The goal of our mini-review was to highlight key studies in the many parallel approaches to mitigating the neuroinflammatory response to brain-dwelling neural electrodes (Table 1). The mini-review was intended as a starting point for discussion towards the integration of a more targeted approach to device integration. As the list of interesting targets within the brain for interfacing continues to grow, a more comprehensive and specific strategy for device integration will be required. Electrode design alone will not be the answer. As a more detailed understanding of the biology at the probe-tissue interface is elucidated, more directed therapeutic interventions which modulate the immune system will be required to improve device performance to a long-term, reliable system. It should be clear that many promising approaches to mitigation of inflammation are under development and studies looking at the effects of neural recording to many of these approaches need to be conducted before the aforementioned approaches are widely adapted. Strategies that utilize immunomodulatory interventions already in the clinic will need to be coupled with responsive local delivery platforms to avoid peripheral anti-inflammatory treatment that should not be a long-term solution. Large animal and clinical demonstration of such approaches will soften the barrier to novel immunomodulatory strategies at earlier stages of development and ultimately bring forth significant benefit to the function of these devices and the rehabilitation of disabled individuals.

## Figures and Tables

**Table 1.** Summary of strategies to mitigate the neuroinflammatory response to intracortical microelectrodes.

Strategy	Approaches	Suggested next steps/remarks
**Broad anti-inflammatory strategies**		
Steroids	Peripheral injections	Development of continuous local delivery platform, determine effect on neural recording
	Probe coatings	
	Electrode site coatings	
	Local delivery via microfludics in probe	
Antibiotics	Add to drinking water	Determine effect local delivery has on neural recording. Other strategies might be less risky.
	Incorporate in thin film coatings	
	Local delivery via microfludics in probe	
	Single local dose via cannula	
**Targets of more specific immunomodulatory strategies**		
Glial Response	Alpha-MSH probe coating	Determine effect on neural recording
	Single dose of flavopiridol	Did not result in improvement in neural recording performance
	IL-IRa probe coating	Determine effect on neural recording
Inflammasome	Caspase knock-out model	Determine effect small molecule or other clinically relevant therapy has or neural recording
Blood-de rived cells	MCP-1 knock out model	Determine effect on neural recording
	Pharmacological inhibition of MCP-1	
Pattern recognition receptors	CD14 knock out model	Determine dosing regimens of small molecule or other clinically relevant therapy
	Pharmacological inhibition of CD14	
